# Atomic description of the immune complex involved in heparin-induced thrombocytopenia

**DOI:** 10.1038/ncomms9277

**Published:** 2015-09-22

**Authors:** Zheng Cai, Serge V. Yarovoi, Zhiqiang Zhu, Lubica Rauova, Vincent Hayes, Tatiana Lebedeva, Qun Liu, Mortimer Poncz, Gowthami Arepally, Douglas B. Cines, Mark I. Greene

**Affiliations:** 1 Department of Pathology and Laboratory Medicine, University of Pennsylvania, Philadelphia, Pennsylvania 19104, USA; 2 Division of Hematology, The Children’s Hospital of Philadelphia, Philadelphia, Pennsylvania 19104, USA; 3 New York Structural Biology Center, National Synchrotron Light Source (NSLS) X4, Brookhaven National Laboratory, Upton, New York 11973, USA; 4 Division of Hematology, Department of Medicine, Duke University School of Medicine, Durham, North Carolina 27710, USA

## Abstract

Heparin-induced thrombocytopenia (HIT) is an autoimmune thrombotic disorder caused by immune complexes containing platelet factor 4 (PF4), antibodies to PF4 and heparin or cellular glycosaminoglycans (GAGs). Here we solve the crystal structures of the: (1) PF4 tetramer/fondaparinux complex, (2) PF4 tetramer/KKO-Fab complex (a murine monoclonal HIT-like antibody) and (3) PF4 monomer/RTO-Fab complex (a non-HIT anti-PF4 monoclonal antibody). Fondaparinux binds to the ‘closed’ end of the PF4 tetramer and stabilizes its conformation. This interaction in turn stabilizes the epitope for KKO on the ‘open’ end of the tetramer. Fondaparinux and KKO thereby collaborate to ‘stabilize’ the ternary pathogenic immune complex. Binding of RTO to PF4 monomers prevents PF4 tetramerization and inhibits KKO and human HIT IgG-induced platelet activation and platelet aggregation *in vitro*, and thrombus progression *in vivo*. The atomic structures provide a basis to develop new diagnostics and non-anticoagulant therapeutics for HIT.

## Abstract (web summary)

Heparin-induced thrombocytopenia (HIT) is an autoimmune thrombotic disease with limited treatment options. Here the authors present crystallographic data on the disease-causing immune complex, providing the structural basis for the development of new diagnostic and therapeutic approaches to HIT.

## Graphical abstract

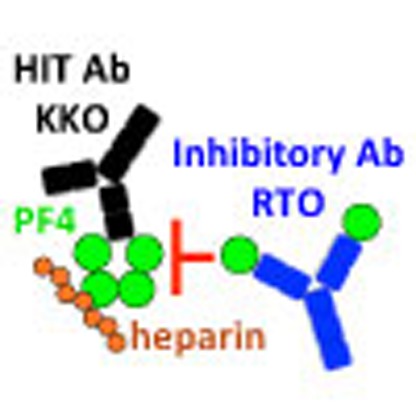

Heparin-induced thrombocytopenia/thrombosis (HIT) is a potentially fatal immune complex mediated thrombotic disorder that develops in ∼1% of patients exposed to unfractionated heparin or, less commonly, to low-molecular-weight heparins^1^. HIT is caused by antibodies to complexes that form between platelet factor 4 (PF4) released from activated platelets and heparin or cellular glycosaminoglycans (GAGs). Circulating immune complexes composed of PF4, heparin and HIT antibodies bind to platelet and monocyte Fcγ receptors and promote cellular activation, leading to generation of thrombin^2^,^3^,^4^. Anti-PF4/heparin antibodies detected by enzyme-linked immunosorbent assay (ELISA) also develop in a high proportion of patients exposed to heparin, for example, after cardiopulmonary bypass surgery, even in the absence of clinical complications. However, the basis for the distinction between pathogenic and non-pathogenic antibodies is unknown, which can lead to overdiagnosis and overtreatment^5^.

In prior studies, we identified a murine monoclonal antibody (KKO) to PF4/heparin complexes that causes heparin-induced thrombosis and thrombocytopenia in a murine model, thus sharing salient features with the clinical disorder^6^. Human HIT antibodies compete with KKO for binding to PF4/heparin, and KKO augments formation of pathogenic immune complexes^7^. An isotype matched anti-PF4 antibody (RTO) that binds comparably to PF4, but does not generate pathogenic complexes^8^, has also been identified.

Here we describe and compare the crystal structures of PF4 in complex with Fabs derived from KKO and RTO and the structure of PF4 in complex with a heparin-mimic pentasaccharide, fondaparinux. These results suggest that by stabilizing the structure of the asymmetric PF4 tetramer, fondaparinux might foster the binding of HIT antibody and formation of the pathogenic ternary complex. Surprisingly, the non-HIT antibody RTO binds to an epitope expressed on the surface of the PF4 monomer that partly overlaps with the KKO epitope in the tetramer. Binding of RTO prevents tetramerization of PF4 and inhibits KKO-induced platelet activation and aggregation *in vitro* and has potent inhibitory effects on thrombus progression *in vivo*.

These crystallographic data lead to a model to help understand the structural basis of the pathogenic immune complex that causes HIT at the atomic level and provide a structural basis for the development of new diagnostics and non-anticoagulant therapeutics for HIT. Our data also provide insights into the process through which a normal human protein becomes ‘antigenic’ to the mammalian immune system after complexing with endogenous or exogenous GAGs.

## Results

### Crystal structure of the fondaparinux/PF4 tetramer complex

Based on previously reported crystal structures, human PF4 can assume an asymmetric tetrameric configuration. Each monomer contains three-stranded antiparallel β-sheets on which an aperiodic N-terminal domain and an amphipathic C-terminal α-helix are folded^9^. NMR studies reveal that PF4 exists as an equilibrium among monomers, dimers and tetramers^10^. The tetrameric structure of PF4 is stabilized not only by salt bridge interactions between dimers AB/CD (hereafter we use the nomenclature for the monomer chains of PF4 as in PDB: 1RHP^9^), but also by the antiparallel β-sheet-like structures in the N termini of dimers AC or BD^9^. It had been assumed that the asymmetry of the PF4 tetramer plays a role in heparin binding^11^ and several models have been proposed to explain the interaction between heparin and PF4 (refs 12, 13, 14). However, conclusive structural data at the atomic level have not been reported.

Binding of pathogenic HIT antibodies to PF4 is markedly enhanced by heparin or various glycosaminoglycans (GAGs), which are themselves highly heterogeneous in size and composition and unsuitable for crystallization. To investigate how the GAGs might induce or augment neoantigen formation, we crystallized PF4 in complex with fondaparinux, a homogenous synthetic pentasaccharide heparin that forms complexes with PF4 as assessed by atomic force microscopy and photon correlation spectroscopy^15^. Fondaparinux induces anti-PF4/heparin antibodies as do other GAGs^16^,^17^,^18^ and can occasionally cause HIT^19^,^20^,^21^,^22^,^23^.

**Figure 1:**
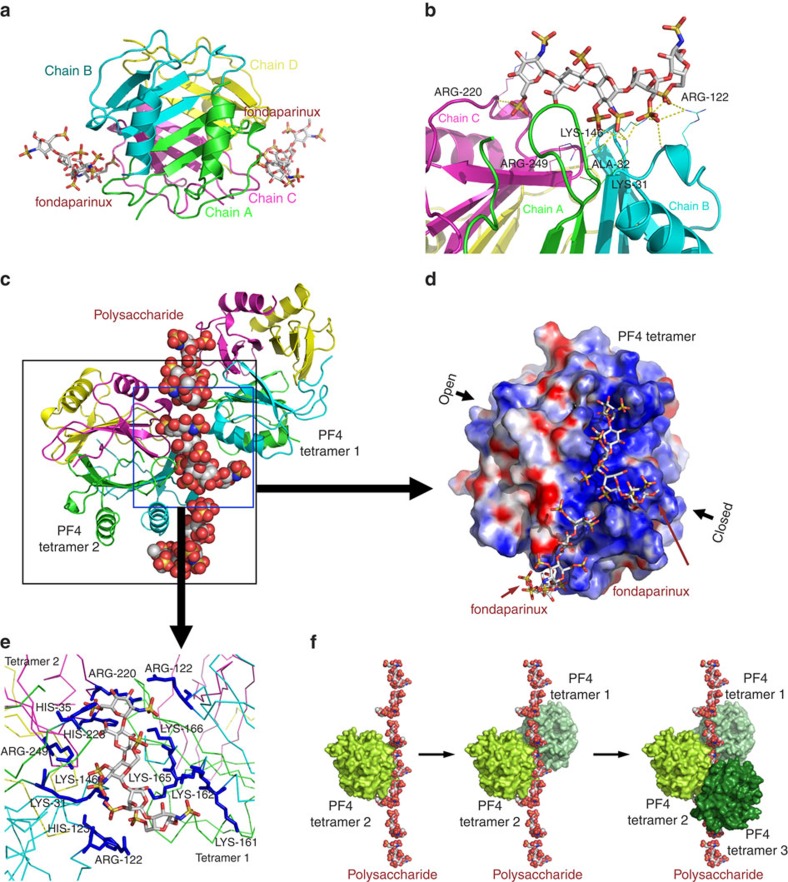
Crystal structure of the fondaparinux/PF4 tetramer complex. (**a**) Overall structure of the PF4/fondaparinux complex. Fondaparinux makes contacts with a single PF4 tetramer in the groove among the monomers on one side of the asymmetric tetramer. Monomers A, B, C and D in one PF4 tetramer are coloured in green, cyan, magenta and yellow, respectively. (**b**) Fondaparinux (stick representations) stabilizes the PF4 tetramer by binding in the groove among three monomers in a PF4 tetramer. Yellow dotted lines indicate the polar interactions between fondaparinux and three PF4 monomers. (**c**) One fondaparinux (spheres) denoted in the blue box binds in the groove of one tetramer (cartoon representation on the left) and also binds to the C-terminal helix of a second tetramer (cartoon representation on the right), thereby bridging PF4 tetramers. (**d**) Electrostatic potential surface representation (positive: blue; negative: red) of the PF4 tetramer shows that fondaparinux binds along a continuous positively charged surface on the ‘closed’ side of PF4 tetramer. (**e**) Detailed representation of the positively charged residues (coloured in blue and labelled) on the fondaparinux binding interface between two PF4 tetramers. (**f**) Analysis of crystal lattice reveals a molecular pathway for the formation of antigenic complexes. A fragment of heparin first binds within the groove of one PF4 tetramer (limon, left); binding of the first PF4 tetramer imparts a local linearized structure on heparin, which enhances the binding of a second tetramer (pale green, middle); progression of this process eventuates in the formation of ultralarge antigenic complexes (right).

We solved the crystal structure of PF4 in complex with fondaparinux as a model GAG. The PF4/fondaparinux complex is a pseudo-222 tetramer formed by a pseudo two-fold non-crystallographic symmetry and a perpendicular two-fold crystallographic symmetry (Fig. 1a, Table 1 for statistics). Fondaparinux appears as a well-defined molecule, evidenced by the strong and clear electron densities (Supplementary Fig. 1). Superposition of the bound and non-bound structures of PF4 tetramer (PDB: 1RHP) using Cα gives a root-mean-square deviation of only 0.76 Å. This indicates that the association with fondaparinux causes minimal conformational changes in PF4 except for slight redirection of side chains around the binding groove, which is consistent with circular dichroism (CD) studies reported recently^24^,^25^.

The PF4 tetramer displays a pseudosymmetry characterized by an ‘open’ end and a ‘closed’ end that can be defined by the intra-chain distances between amino acids E28 and K50. E28 and K50 are ∼3 Å apart and form stable salt bridge pairs between chain A and chain C. However, the same interchain salt bridge pairs do not form between E28 and K50 in chains B and D, which are ∼8 Å apart (Supplementary Fig. 2). This asymmetry in the PF4 tetramer explains why calculation of its electrostatic potential identifies only two positively charged grooves on the ‘closed’ side of PF4 tetramer surface to which fondaparinux can bind (Fig. 1d).

PF4 tetramers can be formed between the AB and CD dimers, or between the AC and BD dimers^9^. Fondaparinux binds among monomers A, B and C or monomers A, C and D (Fig. 1b). This interaction stabilizes the AB/CD association and the AC/BD association, thus further stabilizing the PF4 tetramer. We suggest that this stabilization might represent a very early step in the pathogenesis of HIT antigen.

Analysis of the crystal lattice further reveals that fondaparinux not only makes contact with a single PF4 tetramer within the groove formed by three monomers (ABC or ACD), but also at a second site involving the C-terminal helix of the third monomer (B or D). It has been proposed that the HIT antigen develops when charge neutralization by polyanions allows positively charged PF4 tetramers to undergo close approximation^14^,^15^. The crystal structure of the PF4/fondaparinux complex provides a more complete atomic understanding of this process. One fondaparinux molecule not only binds within the groove on the surface of one PF4 tetramer, it is also shared with a second symmetry-related tetramer through binding to its C-terminal helix (Fig. 1c). Most of these interactions are between the sulfate groups of fondaparinux and the basic residues in PF4 (details in Fig. 1e). The finding that two tetramers of PF4 bind to one heparin fragment provides insight into the initiation of ultralarge complex formation.

Based on what is observed in the crystal, we propose the following pathway for the formation of the heparin/PF4 complex. Heparin first binds to the groove in one PF4 tetramer. Binding of the first PF4 tetramer imparts a local linearized structure on heparin. This enhances the binding of a second tetramer. Progression of this process eventuates in the formation of the large antigenic complex in which PF4 tetramers cluster around a semi-rigid linear heparin chain (Fig. 1f, Supplementary Movie 1). Clustering might be required for apposition of sufficient HIT antibodies to induce persistent activation of cellular FcγIIA receptors^26^. This model provides structural insight into a recently proposed heparin/PF4 interaction model^14^, and is also consistent with previous NMR^27^ and site-directed mutagenesis studies^28^,^29^. Moreover, this set of atomic structures extends other studies showing a heparin chain of ∼10 saccharides is required to form a stable antigenic complex^14^,^25^,^30^,^31^.

### Crystal structure of the KKO-Fab/PF4 tetramer complex

**Figure 2:**
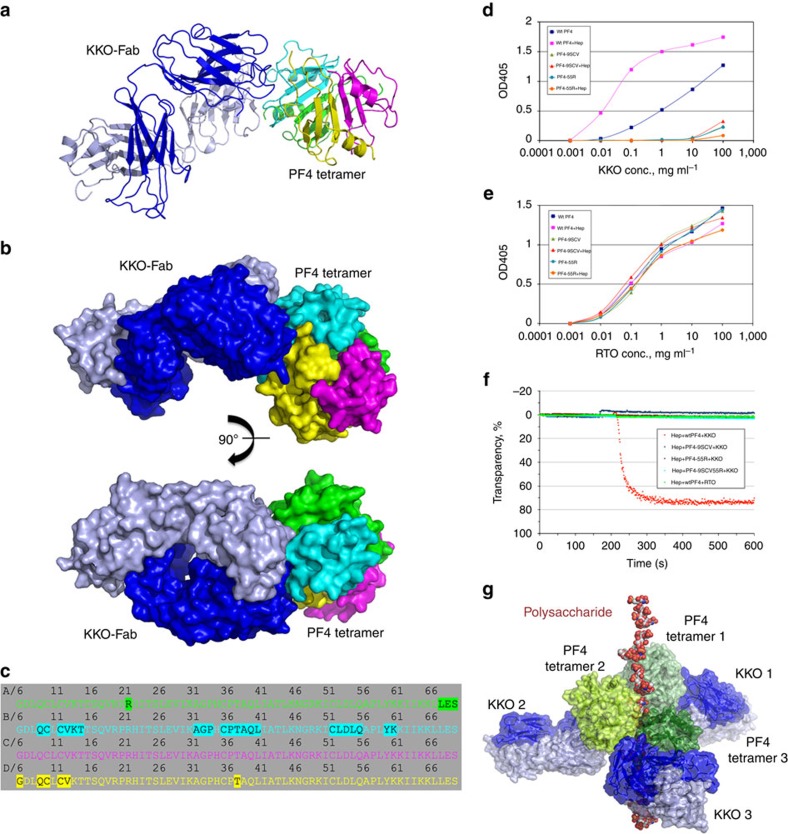
Crystal structure of the KKO-Fab/PF4 tetramer complex. (**a**,**b**) Overall structure of the PF4/KKO-Fab complex. a: cartoon representations of the complex; b: molecular surface representations. The heavy chain and light chain of KKO-Fab are coloured in blue and light blue, respectively. (**c**) Detailed binding interface of HIT antibody KKO to a PF4 tetramer. Residues within a PF4 tetramer that are <5 Å away from KKO-Fab molecule are highlighted. PF4 monomers are coloured as in Fig. 1. (**d**,**e**) Binding of KKO (**d**) and RTO (**e**) to structure-based PF4 mutants. (**f**) Platelet aggregation by wild-type PF4 and PF4 mutants. KKO (red) induced platelet aggregation in the presence of wild-type PF4 and heparin whereas an isotype matched non-pathogenic antibody RTO described below (green) did not. The panel also demonstrates that PF4 mutants bearing mutations along the KKO binding interface (blue, PF4-9SCV; brown, PF4-55R, Cyan, PF4-9SCV55R) were unable to mediate KKO-induced platelet aggregation. (**g**) Model of the KKO-Fab/PF4/heparin ternary complex. Surface representations of KKO-Fab are coloured in blue (heavy chain) and light blue (light chain) and others are coloured as in Fig. 1f. The model assumes the heparin molecule is composed of about 7 structures similar to fondaparinux depicted in the figure as a non-continuous chain. Intact UFH may further enhance the stability of the holo-complex compared with the fondaparinux fragment, thereby rendering it more antigenic and more capable of binding multiple IgG antibodies.

To understand the structural basis for the formation of pathogenic immune complexes, we next solved the crystal structure of human PF4 complexed with the Fab fragment of the monoclonal antibody KKO (Fig. 2a, Supplementary Fig. 7, Table 1 for statistics). KKO recapitulates the salient features of HIT in a mouse model *in vivo* and competes with human platelet activating anti-PF4/heparin antibodies *in vitro*^8^,^26^. The KKO-Fab fragment binds to the PF4 tetramer by making contacts with three monomers (for example, chains A, B and D within one PF4 tetramer, Fig. 2b, surface representations; Fig. 2c, detail of KKO epitope on the PF4 tetramer surface). This structure provides strong support for the concept that formation of the tetramer is required for optimal binding of KKO^32^.

KKO does not bind to murine PF4 (mPF4) complexed with heparin. Therefore, we compared the sequence of human PF4 (hPF4) with mPF4, and made structure-based mutants based on the proposed contact sites identified in the co-crystal (Supplementary Fig. 3). Mutations in the putative KKO epitope essentially abolished binding of the HIT antibody KKO to these variant PF4s by ELISA (Fig. 2d,e) and did not support heparin/KKO-induced platelet activation as measured by light transmission aggregometry (Fig. 2f) in contrast to wild-type PF4 (ref. 6).

### Structural model of the HIT pathogenic ternary complex

Further analysis revealed that PF4 within the PF4/KKO-Fab complex adopts a symmetric tetrameric structure, which is an ‘open’-‘open’, possibly intermediate, conformation (Supplementary Fig. 4). This result enabled us to superimpose the PF4/KKO-Fab complex onto the PF4/heparin complex model (Fig. 1f) to build a structural model of the KKO-Fab/PF4/‘heparin’ ternary complex. In this model, the polysaccharide binds to the ‘closed’ end of PF4, stabilizing the PF4 tetramer, which orients the ‘open’ end for recognition by KKO. In this way, fondaparinux and HIT-like antibody collaborate to ‘stabilize’ the ternary complex (Fig. 2g, Supplementary Movie 2).

These structures provide the first atomic level description of the pathological complex that might occur in HIT. The model might also help to explain why fondaparinux, a short fragment of heparin, which is antigenic, only rarely causes HIT. Although the protein–GAG interactions can in theory be propagated to additional PF4 tetramers and fondaparinux molecules, we propose that covalent bonding between pentasaccharide units in longer heparins markedly increases the stability of the holo-complex. Consequently, the more ‘stable’ complex becomes more antigenic by enhancing antibody avidity.

### Crystal structure of the RTO-Fab/PF4 monomer complex

RTO is an anti-PF4 antibody that possesses the same isotype as KKO, but does not cause HIT *in vivo*^6^,^33^. In contrast to KKO, binding of RTO to hPF4 is not enhanced by heparin^6^. RTO and KKO also do not compete for binding to hPF4 by ELISA^7^. However, KKO and RTO display the same Bmax when measured by ELISA, and is thus illustrative of the difficulty in distinguishing human pathogenic and non-pathogenic antibodies using contemporary bulk-equilibrium assays^7^. The reason why RTO does not cause HIT was revealed through atomic analyses.

**Figure 3:**
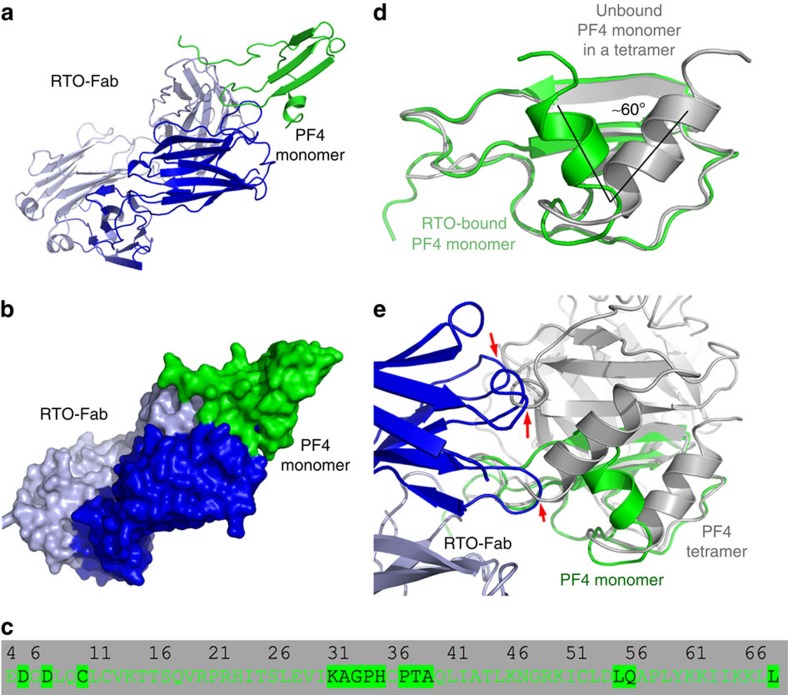
Crystal structure of the RTO-Fab/PF4 monomer complex. (**a**,**b**) Overall structure of the PF4/RTO-Fab complex. a: cartoon representations of the complex; b: molecular surface representations. The heavy chain and light chain of RTO-Fab are coloured in blue and light blue, respectively. (**c**) Detailed binding interface of the non-HIT antibody RTO to a PF4 monomer. Residues of a PF4 monomer that are <5 Å away from RTO-Fab molecule are highlighted. (**d**) Superposition of the PF4 monomer (green) in the RTO-Fab/PF4 complex with that in the unbound PF4 (grey) indicates that binding of RTO-Fab causes a dramatic structural change in the PF4 monomer: the C-terminal helices are shifted ∼60°. (**e**) Superposition of the PF4 monomer (green) in complex with RTO-Fab (blue and light blue) with the unbound PF4 tetramer (grey). The three arrows indicate the sites where binding of RTO-Fab to one PF4 monomer causes steric clashes with a second PF4 monomer in the tetramer, thereby preventing tetramer formation.

To help understand the difference between the impacts of these two antibodies, we next solved the crystal structure of the RTO-Fab in complex with hPF4 (Fig. 3a,b, Supplementary Fig. 7, Table 1 for statistics). Unexpectedly, RTO-Fab binds only to the PF4 monomer. This was affirmed by a change in the migration of PF4 monomer, but not PF4 tetramer, upon addition of RTO-Fab assessed by fast protein liquid chromatography (Supplementary Fig. 5).

Also unexpectedly, the RTO epitope on PF4 overlapped considerably with the KKO epitope (compare Fig. 3c with Fig. 2c, Supplementary Fig. 6). In the structure of the RTO-Fab/PF4 complex, the C-terminal helices still pack against the β-sheet domain. However, the orientation of the C-terminal helices shifts ∼60° relative to the β-sheet core domain (Fig. 3d). Superimposition of the PF4 asymmetric tetramer onto the RTO-Fab/PF4 complex reveals that RTO binds between monomer A and monomer B and prevents the formation of the AB dimer, and thereby prevents formation of the tetramer (formation of an AC dimer is still theoretically possible; Fig. 3e). Together, these structural data lead us to propose that tetramerization of PF4, which is required to form the HIT antigen, would be disrupted by the non-HIT antibody RTO.

### RTO limits tetramer formation preventing platelet activation

**Figure 4:**
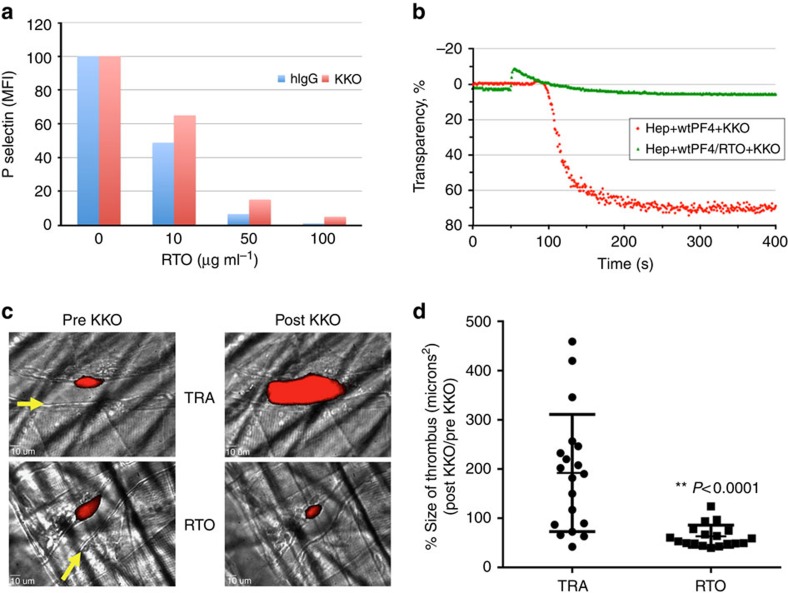
RTO prevents KKO-induced platelet activation and thrombosis. (**a**). Inhibition of KKO/PF4 mediated platelet activation by RTO. Samples of whole blood were incubated with the indicated concentrations of RTO in the presence of PF4 (10 μg ml^−1^) for 15 min before adding the platelet-activating anti-PF4 antibody KKO or human IgG. Activation of platelets were followed by expression of P-selectin; the effect of RTO is expressed as % of geometric mean fluorescent intensity (MFI) of P-selectin expression on platelets relative to MFI in the absence of RTO. (**b**) *In vitro* platelet activation assay demonstrated that pre-incubation of PF4 with RTO prevented KKO-induced platelet aggregation. (**c**) Representative composite images of platelet fluorescence overlaid on brightfield snapshots of injuries in mice receiving either RTO or the IgGk2B isotype control TRA are shown. Pre KKO images show thrombi 15 min after initial injury and injection of RTO or TRA. Post KKO images represent the same thrombus 15 min after KKO had been injected intravenously. Arrows represent the direction of blood flow. (**d**) Each dot denotes the per cent change in the size of a single injury based on binding of fluorescently labelled platelets in mice receiving either RTO or the IgGκ2B isotype control TRA followed by KKO. Error bars show the standard deviation. *N*=18 injuries in three mice for RTO, *N*=19 injuries in three mice for TRA. *P*<0.0001.

Based on the knowledge that PF4 tetramers exist in equilibrium with monomers and dimers, and with even higher ordered complexes when heparin is present, we predicted that RTO might prevent or disrupt tetramer formation and thereby prevent KKO-induced platelet activation in the presence of heparin. Indeed, RTO inhibited the activation of platelets by KKO and human HIT IgG in the presence of heparin in a dose-dependent manner, as assessed by expression of P-selectin on flow cytometry (Fig. 4a). Pre-incubation of PF4 with RTO also inhibited KKO-induced platelet aggregation (Fig. 4b).

Moreover, RTO completely inhibited KKO-induced progression of thrombosis in a laser microvascular injury model in mouse PF4^−/−^/human PF4^+/+^/human FcRγIIA^+^ mice^26^ (Fig. 4c, d). These data indicate that the non-HIT antibody RTO, when engineered and humanized, could provide a template for the development of non-anticoagulant intervention in HIT.

## Discussion

It is uncertain how heparin or an endogenous GAG converts a normal host protein, PF4, into an ‘autoantigen’. Our crystallographic data provide insight into the multistep process of neoantigen formation at the atomic level. We report the first crystal structure of the PF4 monomer, here in complex with the Fab fragment of an anti-PF4 antibody RTO, which affirms that PF4 exists in solution as an equilibrium among monomers, dimers and tetramers^10^. In the monomeric state, the N-terminal amino acids of PF4 adopt a flexible extended conformation and the C-terminal helices swing around atop the β-sheet core domain. In contrast, in the tetrameric state the individual monomers undergo a marked conformational change. The C-terminal helices of the PF4 monomers are all reorientated and realigned and the N termini of the monomers form antiparallel β-sheet-like structures. The repulsive forces between the positively charged C-terminal helices of monomer A and monomer B in the AB dimer (and similarly between monomer C and monomer D in the CD dimer) might induce thermal instability in the tetramer^34^. Heparin binds among PF4 monomers and also bridges PF4 tetramers, which helps overcome this instability and leads to the formation of stable individual tetramers, a fundamental first step in the expression of the neoantigen in HIT.

Our data also indicate that the asymmetry of the fondaparinux-bound PF4 tetramer, which has an ‘open’ end and a ‘closed’ end, plays an important role in forming the pathogenic antigenic complex. Based on our findings, we propose that heparin or a GAG fragment stabilizes the PF4 tetramer by clamping the monomers together through the ‘closed’ end, orienting the ‘open’ end in the asymmetric PF4 tetramer that comprises the binding site for KKO. Thus, in the absence of heparin, the KKO binding site on the tetramer is nonexistent or incompletely expressed on the PF4 monomer and dimer and is only transiently expressed and unstable on tetramers that form at high concentrations of PF4. Thus, stabilization of the tetramer by heparin leading to a more stable orientation of an epitope on each PF4 tetramer, compared with the transient and more random orientation of the epitope in the absence of heparin, is a second defining step in the process of generating the HIT neoantigen. Heparins or GAGs of sufficient lengths can then cluster several PF4 tetramers further enhancing stability, which helps to explain why PF4, among heparin binding proteins, is especially antigenic^7^,^14^. In the third step, HIT-like antibody KKO recognizes the stabilized ‘open’ end of the PF4 tetramers, further promoting formation of the pathogenic ternary complex.

Our studies were performed with fondaparinux because the heterogeneity of clinical heparins and GAGs preclude obtaining structural detail. Therefore, we do not exclude the possibility that low molecular weight or unfractionated heparin might induce additional changes within and between PF4 tetramers^24^,^35^. Indeed, our structural model predicts that when a GAG longer than about 10 saccharides binds to more than one PF4 tetramer, additional structural changes within the tetramers themselves are quite likely. These structural changes are more likely to occur within the inner surface of the PF4 tetramers in the cluster along the PF4/heparin binding interface than primarily at the HIT-antibody binding epitope, which lies on the outer surface. Therefore, additional studies are needed based on this model, to determine if these additional structural changes in PF4 imparted by longer GAGs directly modulate the contact sites between antibody and antigen.

**Figure 5:**
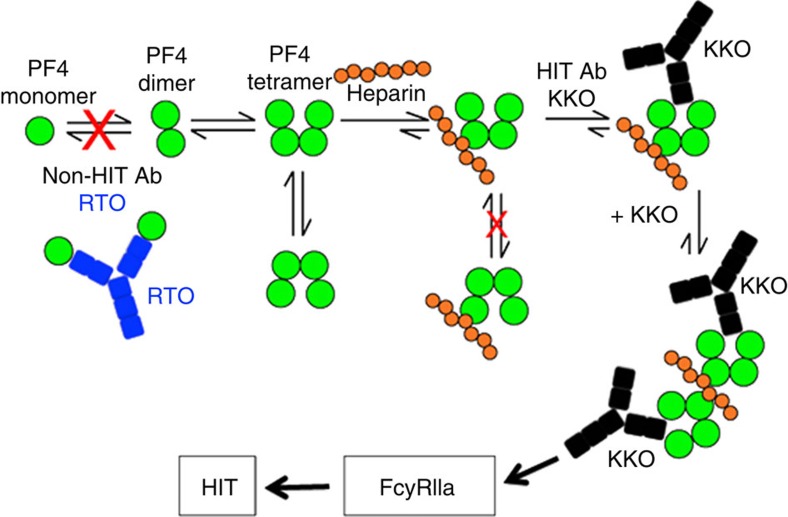
Model of RTO inhibition of heparin-induced thrombocytopenia. PF4 molecules (green circles) exist in an equilibrium among monomers, dimers and tetramers. On binding to heparin (orange circles), the configuration of the tetramer is stabilized. As a result, the open end of the PF4 tetramer is oriented and recognized by HIT-antibody KKO (black). KKO in turn fosters PF4 oligomerization and works collaboratively with heparin to stabilize the ternary complex. The net result is the generation of stable ultralarge immune complexes capable of sustained activation of Fcγ receptors on platelets and monocytes, which consequently leads to HIT. The non-HIT isotype-matched antibody RTO (blue) binds to PF4 monomers, prevents PF4 oligomerization, prevents formation of ultralarge immune complexes and as a result may prevent HIT. The cartoon assumes heparin is composed of about seven structures similar to fondaparinux.

The A32-A39 loop on the PF4 monomer appears to be highly immunogenic. However, our data suggest that pathogenic and non-pathogenic antibodies that bind to this region differ in how they affect the monomer–dimer–tetramer equilibrium. Pathogenic antibodies that act like KKO and preferentially recognize the tetrameric species display greater avidity in the presence of heparin, which approximates epitopes, and in turn the bound antibody fosters oligomerization^7^. Binding of KKO may also shift the equilibrium from heparin wrapping around the PF4 species suggested in a previous model^13^ by forcing it into a more stable linear conformation^14^. The net result would be the generation of stable ultralarge immune complexes^36^ capable of sustained activation of FcγIIA receptors on platelets and monocytes^26^,^37^. In contrast, anti-PF4 antibodies that preferentially recognize monomers in the manner of RTO are readily detected by contemporary ELISAs, but they do not generate large immune complexes that cause disease. Indeed, anti-PF4 antibodies that act like RTO may actually compete with pathogenic antibodies by preventing or disrupting tetramer formation and thereby limit formation of larger immune complexes as depicted in Fig. 5. These data provide a new mechanistic model for the development of a human autoimmune disease, in which a host-protein PF4 complexed with host GAGs assumes diverse oligomeric conformations that differentially bind autoreactive antibodies leading to diverse clinical outcomes.

Our studies might have implications for the diagnosis of HIT. The two prototypic antibodies we studied, RTO and KKO, bind comparably to PF4/heparin on ELISA plates at equilibrium and do not compete with each other for binding in this format^7^,^8^. This suggests that ELISA wells might contain PF4 in diverse conformations ranging from monomer through tetramer and likely higher ordered complexes. Studies are in progress to determine if ELISA formats based on more homogenous populations of PF4 complexes and/or PF4 variants will reduce detection of non-pathogenic, potentially ‘blocking’, anti-PF4 antibodies. Additional studies will also be needed to determine if differences in the ratio of pathogenic and ‘blocking’ antibodies might contribute to the likelihood of developing HIT.

Our studies might also have implications for therapy. Anticoagulants are the standard treatment for HIT, but their efficacy is incomplete and dosing is limited by the risk of bleeding^5^,^38^. Our data indicate that RTO binds to PF4 monomers and preempts assembly of stable tetramers and, as a result, inhibits KKO (and human HIT IgG) induced platelet activation and aggregation *in vitro* and, importantly, progression of antibody-induced thrombosis *in vivo*. It is likely release of PF4 and formation of antigenic complexes extend beyond diagnosis and introduction of a direct thrombin inhibitor, as does the risk of recurrent thromboembolic complications. The inhibitory effects of RTO indicate that tetramerization of PF4 is targetable and that this antibody may provide a structural basis for developing rational non-anticoagulant HIT-specific intervention for this serious and common iatrogenic disorder.

It has been difficult to differentiate between the impact of changes within the secondary structure of individual PF4 tetramers that affect antibody affinity and effects on tetramer oligomerization that enhance avidity, because both contribute to antibody binding measured in ELISA wells that likely contain various conformations of PF4. Our use of Fab fragments that are unable (or show little ability) to oligomerize PF4 tetramers, and fondaparinux, which is antigenic but rarely pathogenic because it has a low capacity to form higher ordered structures, provided an opportunity to delineate the evolution of the antigenic site within the PF4 tetramer itself.

Nevertheless, we wish to emphasize that our studies also have potential limitations. First, the necessity of using fondaparinux as the GAG precluded possible additional relevant changes within PF4 induced by longer heparin molecules that are more likely to induce HIT. Nor can we assess the intra-molecular and intermolecular effects on PF4 imparted by the complex array of cellular GAGs. Second, although most HIT antibodies compete with KKO for binding, our data do not preclude additional contact sites between polyspecific human antibodies and PF4/heparin. Third, crystallography and other biophysical approaches using purified proteins inherently explore atomic interactions in a single stable structure favored by the experimental conditions. Thus, the full range of dynamic changes in (super)oligomerization that occur during the evolution of the disease may not be captured. Thus, our study only provides a model that may help to explain sentinel events through which GAGs may induce binding of autoantibodies to a normal host protein. Fourth, in our experiments, RTO was added before KKO. The dynamics of RTO binding to PF4 might well be different when heparin and a pathogenic HIT antibody are already present. We are currently investigating the effect of RTO on thrombus growth *in vivo* following exposure to KKO.

## Methods

### Expression and purification of human PF4 and antibodies

Wild-type hPF4 and hPF4 mutants in plasmid pMT/BiP/V5-His (Invitrogen Corp., Carlsbad, CA), were expressed using the Drosophila Expression System (Invitrogen), purified and characterized^39^. Briefly, the protein was collected in serum-free medium Insect-Xpress (Lonza, Walkersville, MD) and isolated by affinity chromatography using a HiTrap Heparin HP column (GE Healthcare) on an AKTA Purifier (GE Healthcare) at 4 °C and eluted at 1.8 M NaCl (wtPF4) using a linear gradient. Fractions containing purified PF4 detected by silver staining of 12% polyacrylamide gels (SDS–polyacrylamide gel electrophoresis ) were pooled, concentrated and buffer exchanged into 50 mM HEPES, 0.5 M NaCl, pH∼7.2 using an Amicon Ultra filter (3,000 molecular weight cutoff, Millipore). Protein was quantified using a BCA assay (Pierce). To obtain the PF4 mutants, PCR with corresponding primers (Supplementary Table 1) was performed on pMT/BiP/V5/His-PF4 plasmid under conditions recommended by The QuikChange Site-Directed Mutagenesis Kit manual (Stratagene, La Jolla, CA). The resulting plasmids were sequenced to confirm the mutation.

The murine anti-human PF4 IgG2bκ monoclonal antibodies KKO and RTO have previously been described^6^. The IgGs were purified from conditioned PFHM-II media (Invitrogen) using protein A-agarose (Invitrogen) as recommended by the manufacturer. IgG purity was demonstrated by SDS–polyacrylamide gel electrophoresis on NuPAGE 4–12% Bis-Tris Gel (Invitrogen). Fab fragments were generated by papain digest using Pierce Fab Preparation Kit (Thermo Scientific, Rockford, IL) essentially as recommended by the manufacturer, followed by three rounds of removing of Fc fragments with protein A-agarose beads, and extra purification with anti-mouse IgG (Fc-specific) (Sigma M4280) and anti-mouse IgG (Fab-specific) Sigma M4155 antibodies bound to CNBr-activated Sepharose 4 Fast Flow beads (Amersham Biosciences Corp., Piscataway, NJ) as recommended by the manufacturer. KKO-Fab, RTO-Fab and PF4 were further purified by size-exclusion column on an AKTA purifier system (GE Healthcare). Human HIT IgG was purified using staph protein agarose (source) from a pheresate obtained from a patient with HIT.

### ELISA assays

Binding of human IgG was measured essentially as previously described for KKO and RTO antibodies^7^. Briefly, Immulon 4 HBX 96-well plates (Thermo Fisher Scientific, Waltham, MA) were coated overnight with either PF4 or PF4 mutant at 5 ug ml^−1^. The plates were incubated for 30 min with either PBS (control) or with 0.5% glutaraldehyde at room temperature, extensively washed and blocked with 1% bovine serum albumin (BSA) in PBS. The plates were incubated with human patient IgG samples at experimentally selected concentration of 20 μg ml^–1^ for 30 min at 37 °C. IgG binding was measured as absorbance at 405 nm (A405) after incubation for 30 min at 37 °C with horseradish peroxidase-conjugated ImmunoPure Goat Anti-Human IgG (H+L), HRP Conjugated Product No. 31412 (Pierce. Rockford, IL) diluted 1:10,000 in 1% BSA/PBS. Horseradish peroxidase substrate ABTS was from Roche Applied Science, Penzberg, Germany. Absorbance was measured with a SpectraCount plate reader (Packard BioScience, Waltham, MA).

### *In vitro* platelet activation mediated by KKO+PF4

Blood for *in vitro* studies (platelet activation and light transmission aggregometry) was collected after informed consent from healthy, aspirin-free volunteers using a 19-gauge butterfly needle in 129 mM sodium citrate (10:1, vol/vol) under protocols approved by the Institutional Review Board of the University of Pennsylvania and the Children’s Hospital of Philadelphia. Whole blood samples were incubated in Ca^++^/Hepes buffer (2.5 mM CaCl_2_, 1.25 mM MgCl_2_, 150 mM NaCl, 10 mM HEPES, pH 7.5) 1/100 v/v in the presence of allophycocyanin (APC) labelled anti-hCD41 and PE labelled anti-P-selectin, PF4 (10 μg ml^−1^) and the concentrations of RTO MOAb indicated in the figure for 15 min at room temperature. KKO (20 μg ml^−1^) or human HIT IgG (500 μg ml^−1^) was added for 20 min at room temperature, samples were then diluted by adding 400 μl of HBSA/BSA/EDTA buffer and immediately measured by flow cytometry (BD LSRFortessa). Platelets were gated based on the forward-scatter and CD41 fluorescence parameters, and binding of anti-P-selectin antibodies was quantified as geometric mean fluorescent intensity.

### Light transmission aggregometry

Blood was centrifuged for 12 min at 210*g* at 25 °C to generate platelet-rich plasma (PRP) and at 900*g* for 10 min at 25 °C to produce platelet-poor plasma. Platelet aggregation was measured in PRP using a dual-channel lumi-aggregometer (model 700, Chrono-log Corporation, Havertown, PA) per the manufacturer’s instructions. All experiments were completed within 4 h of blood collection. PRP (500 μl) was pre-warmed for 2 min at 37 °C, heparin (Sagent Pharmaceuticals, Schaumburg, IL) was added (final concentration 0.1 U  ml^−1^) for 30 s followed by wild type or mutant rPF4 (final concentration10 μg ml^−1^) for 30 s followed by KKO or RTO (100 μg ml^−1^) for up to 10 min. The final volume of added reagents did not exceed 5% of the starting volume of PRP. To examine inhibition of KKO-induced platelet aggregation, 100 μg RTO was preincubated with 5 μg PF4 for 15 min at 25 °C. Heparin (final concentration 0.1 U  ml^−1^) was added to pre-warmed PRP. After 30 s PF4 or PF4/RTO complex was added (final concentrations in PRP 10 μg ml^−1^ and 10/200 μg ml^−1^, respectively). KKO (final concentration of 100 μg ml^−1^) was added 30 s later and aggregation was assessed as above.

### Inhibition of KKO-induced thrombosis *in vivo*

Transgenic male C57BL mice, lacking mouse PF4 but expressing human PF4 and human FcγRIIA^26^ were studied. Mice were matched littermates between 6 and 10 weeks of age. The cremaster laser injury model^33^,^40^ was used to visualize *in vivo* thrombus formation. After surgical preparation, Alexa 647 (BD Biosciences) labelled mouse CD41-F(ab’)_2_ fragments were infused intravenously to label circulating platelets. Each mouse then received 50 μg g^−1^ of either RTO or the IgGκ2B isotype control TRA intravenously followed by 7–8-focal arterial injuries. A brightfield and fluorescence snapshot of each injury was taken 15 min after the initial injury. KKO was then injected intravenously at a dose of 2.5 μg g^−1^. Fifteen minutes after injection of KKO, a second brightfield and fluorescence snapshot of the same injuries was taken to compare platelet deposition before and after KKO. Image analysis was performed using the Slidebook 6 (3I Intelligent Imaging Innovations, Denver, CO). The size of the thrombus was determined by having the software automatically segment the image using Otsu thresholding to remove background and then calculate and export the size of the platelet fluorescence in microns^2^. The investigator was not blinded during these studies. All experiments were performed in compliance with the institutional guidelines for the care and handling of experimental animals approved by the Children’s Hospital of Philadelphia Institutional Animal Care and Use Committee.

### Statistical analysis

Statistical analysis of *in vivo* injuries was performed using the Prism 6 (GraphPad, La Jolla, CA). A two-tailed Student’s *t*-test under non-parametric conditions with Mann–Whitney correction was performed to assess statistical significance. *P* values<0.05 were considered significant. There was no randomization of mice. The mice used were selected based on availability. There was no exclusion criteria established before experimentation, available healthy mice were selected for use. No tests of normality or power analyses were conducted.

### Crystallization data collection

Purified KKO-Fab (5 mg ml^−1^) and RTO-Fab (5 mg ml^−1^) were mixed with hPF4 at different ratios and incubated on ice overnight before setting up crystallization trials. The hPF4/fondaparinux complex was prepared by adding six-fold molar excess fondaparinux (Arixtra, Sanofi-Synthelabo LLC) into purified hPF4 (7 mg ml^−1^); the mixture was incubated on ice in a buffer containing PBS and 0.3 M NaCl.

Crystallizations of hPF4/fondaparinux, KKO-Fab, hPF4/KKO-Fab and hPF4/RTO-Fab were performed using the hanging-drop vapour diffusion method by mixing the protein and well solution at 1:1 volume ratio at 16 °C. Crystallization kits from Hampton Research and Molecular Dimensions were used for initial crystallization trials. Optimized hPF4/fondaparinux complex crystals were obtained in the well solution containing 2% PEG4000, 17% MPD and 0.1 M sodium acetate pH 5.6. hPF4/fondaparinux complex crystals were then directly flash-cooled in liquid nitrogen by using the MiTeGen micromounts (MiTeGen, LLC). Diffraction-quality KKO-Fab crystals were obtained in the well solution containing 14% PEG2000, 0.06 M zinc acetate and 0.1 M sodium cacodylate, pH 6.8. KKO-Fab crystals were transferred into the well solution supplemented with 25% glycerol, soaked for one second, and then flash-cooled in liquid nitrogen. Diffraction-quality hPF4/KKO-Fab crystals were obtained in the well solution containing 7% PEG6000, 0.1 M Tris-HCl, pH 7.8. hPF4/KKO-Fab complex crystals were flash-cooled in liquid nitrogen similarly as KKO-Fab crystals. hPF4/RTO-Fab complex crystals were obtained in the well solution containing 0.2 M ammonium sulfate, 0.1 M BIS-TRIS, pH 6.5, 25% w/v polyethylene glycol 3350. The diffraction-quality hPF4/RTO-Fab crystals were optimized by macro-seeding. hPF4/RTO-Fab complex crystals were transferred into the well solution supplemented with 20% glycerol, soaked for one second, and then flash-cooled in liquid nitrogen.

All crystallographic data sets were collected at 100 K with ADSC CCD detectors. The long-wavelength (λ=2.07 Å) sulfur anomalous diffraction data sets for hPF4/fondaparinux complex and hPF4/RTO-Fab complex were collected at beamline X4A at National Synchrotron Light Source at Brookhaven National Laboratory (Upton, NY USA). Other data sets were collected at National Synchrotron Light Source beamlines X6A and X4C at Brookhaven National Laboratory. All diffraction data were processed by using the HKL-2000 package^41^.

### Structure determination and refinement

All structures were solved by molecular replacement. The structure of the hPF4/fondaparinux complex was solved by the CCP4 program MOLREP^42^,^43^ with AB dimer from the hPF4 structure (PDB ID code:1F9Q) as a search model. The structure of KKO-Fab was also solved by MOLREP with the Fab structure from the DsbB-Fab complex (PDB ID code:2ZUQ) as a search model. The structure of the hPF4/KKO-Fab complex was solved by the CCP4 program Phaser^44^ with the refined KKO-Fab structure and hPF4 (PDB ID code: 1RHP) structure as search models. The structure of the hPF4/RTO-Fab complex was solved by Phaser using the structure of 2H2 Fab fragment of immature Dengue virus (PDB ID code:4KVC) and the structure of the A chain of the hPF4 monomer (PDB ID code: 1F9Q) as search models. The final solution has 8 hPF4/RTO-Fab complexes, related by non-crystallographic symmetry, in an asymmetric unit.

All models were iteratively built in COOT^45^ and refined by REFMAC^46^,^47^ or PHENIX^48^. Refinement of the hPF4/KKO-Fab complex at low resolution was performed using the deformable elastic network (DEN)-assisted refinement^49^, REFMAC and OPUS-XREF^50^. To assist model building, the low resolution electron density maps for the hPF4/KKO-Fab complex were optimized using B-factor sharpening. The anomalous diffraction data colleted at long wavelength were used to assist model building and refinement of the hPF4/fondaparinux and hPF4/RTO-Fab complexes. The quality of refined models was checked by program PROCHECK^51^ and MOLPOBITY^52^. All structural figures were prepared in PyMol ( http://www.delanoscientific.com/). The electrostatics potentials were calculated by program APBS, an adaptive Poisson–Boltzmann solver^53^,^54^. Data collection and refinement statistics are listed in Supplementary Table S1.

## Additional information

**Accession codes:** Atomic coordinates and structure factor files have been deposited in the Protein Data Bank (PDB) under the accession codes 4R9W for hPF4/fondaparinux complex, 4R97 for KKO-Fab, 4R9Y for hPF4/KKO-Fab complex, 4RAU for hPF4/RTO-Fab complex.

**How to cite this article**: Cai, Z. *et al*. Atomic Description of the Immune Complex involved in heparin-induced thrombocytopenia. *Nat. Commun.* 6:8277 doi: 10.1038/ncomms9277 (2015).

## Supplementary Material

Supplementary Information Supplementary Figures 1-7 and Supplementary Table 1

Supplementary Movie 1 A three dimensional view of the Heparin/PF4 antigenic complex model

Supplementary Movie 2 A three dimensional view of the Heparin/PF4/KKOFab ternary immune complex model

## Figures and Tables

**Table 1:** Summary of crystallographic data and refinement statistics.

	**PF4/Fondaparinux**	**KKO-Fab**	**KKO-Fab/PF4 tetramer**	**RTO-Fab/PF4 monomer**
*Data collection*				
Beamline	X4A/X4C(BNL)	X6A(BNL)	X4A/X4C(BNL)	X4A/X4C(BNL)
wavelength (Å)	0.9791	0.9795	0.9792	2.07
Space group	P3_2_21	P2_1_2_1_2_1_	P2_1_2_1_2_1_	P2_1_2_1_2
Cell dimensions	*a*=67.33 Å	*a*=41.48 Å	*a*=49.49 Å	*a*=161.42 Å
	*b*=67.33 Å	*b*=92.12 Å	*b*=99.34 Å	*b*=171.87 Å
	*c*=61.76 Å	*c*=122.14 Å	*c*=261.74 Å	*c*=208.16 Å
	α=β=90°, γ=120°	α=β=γ=90°	α=β=γ=90°	α=β=γ=90°
Resolution* (Å)	2.50 (2.54–2.50)	2.20 (2.28–2.20)	4.10(4.17–4.10)	3.75 (3.81–3.75)
Rsym or Rmerge	0.057(0.72)	0.088(0.66)	0.089(0.44)	0.154(1.65)
*I*/*σI*	22.6(3.0)	10.8(2.2)	7.9(1.9)	6.8(2.9)
Completeness (%)	98.2(98.6)	94.4(98.5)	87.9(61.3)	99.4(98.9)
Redundancy	10.3(10.0)	5.1(4.9)	8.9(4.5)	26.5(25.6)
*Refinement*				
Resolution(Å)	22.76–2.50	30.8–2.20	20–4.11	50–3.74
No.unique reflections	5,913	24,599	10,642	60,619
*R*_work_/*R*_free_	0.224/0.245	0.200/0.262	0.317/0.382	0.257/0.297
No. atoms				
Protein	973	3,322	8,548	30,074
Solvent	17	99	0	0
B-factors(Å^2^)				
Protein	95	37	268	134
ligand	157	NA	NA	NA
r.m.s deviations				
Bond lengths(Å)	0.006	0.008	0.006	0.007
Bond angles (°)	0.93	1.17	0.92	1.34
Ramachandran(%)				
Favored/disallowed	100/0	99.4/0.3	97.1/0.8	97.4/1.0

NA, not applicable.

^*^Values in the outermost shell are given in parentheses.
