# Supplementary material for: Atomic description of the immune complex involved in heparin-induced thrombocytopenia
Source: Nat Commun. 2015 Sep 22;6:8277. doi: 10.1038/ncomms9277 (PMC4580983; doi:10.1038/ncomms9277)
Supplement: Supplementary Information — Supplementary Figures 1-7 and Supplementary Table 1 [file ncomms9277-s1.pdf]

Supplementary Figure 1. Well-defined Fondaparinux electron density.

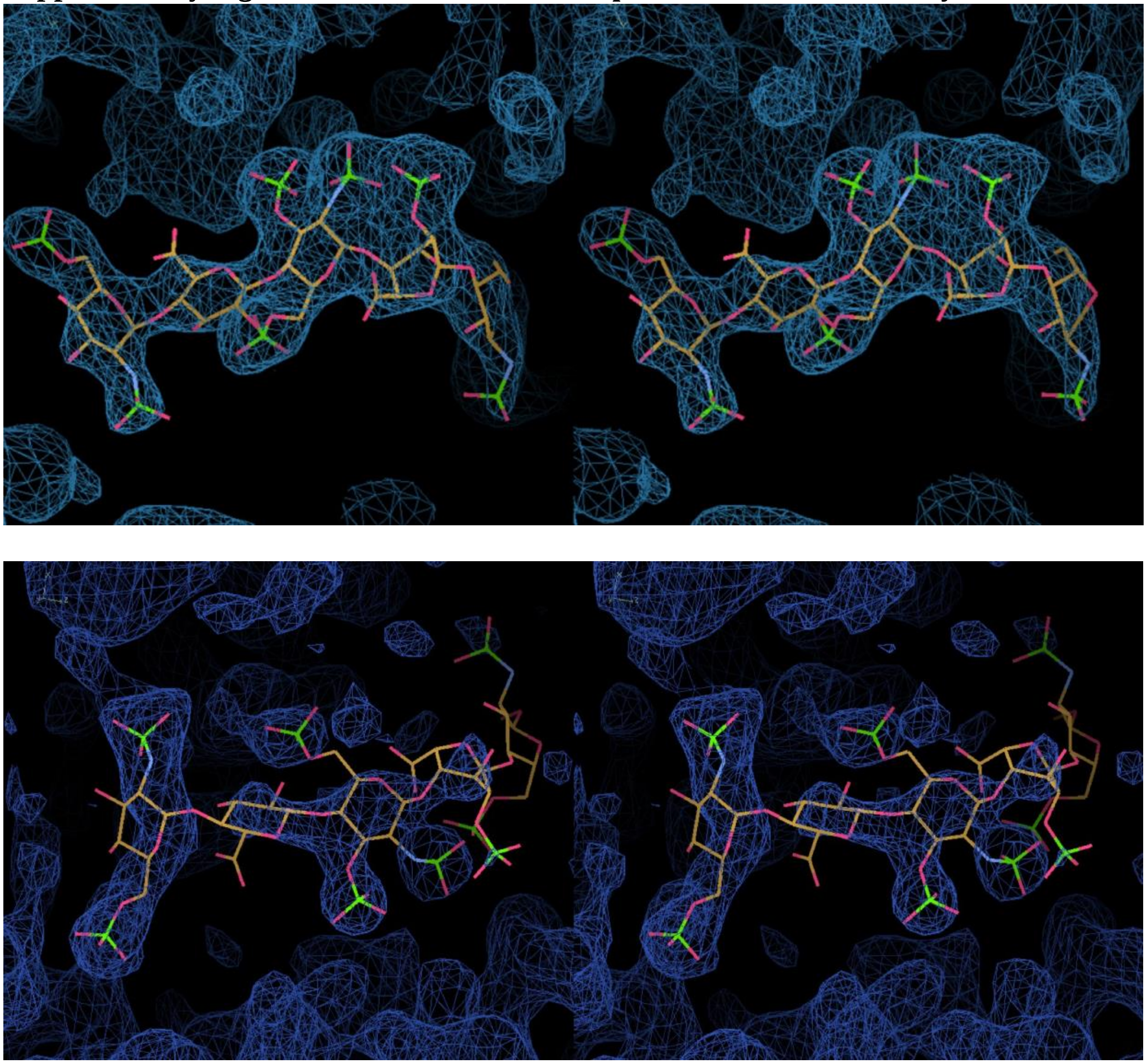

Up: Stereo view of the 2Fo−Fc electron density map (at 1.4σ contour level and colored in blue) fitted with a fondaparinux in the PF4/fondaparinux complex. Down: the 2Fo-Fc OMIT electron density in the fondaparinux binding site, contoured at 1.4 σ. The positions of the ordered sulfate groups are confirmed by the anomalous signals of sulfur.

Supplementary Figure 2. The asymmetric 'open'-'closed' conformation in the PF4/fondaparinux complex.

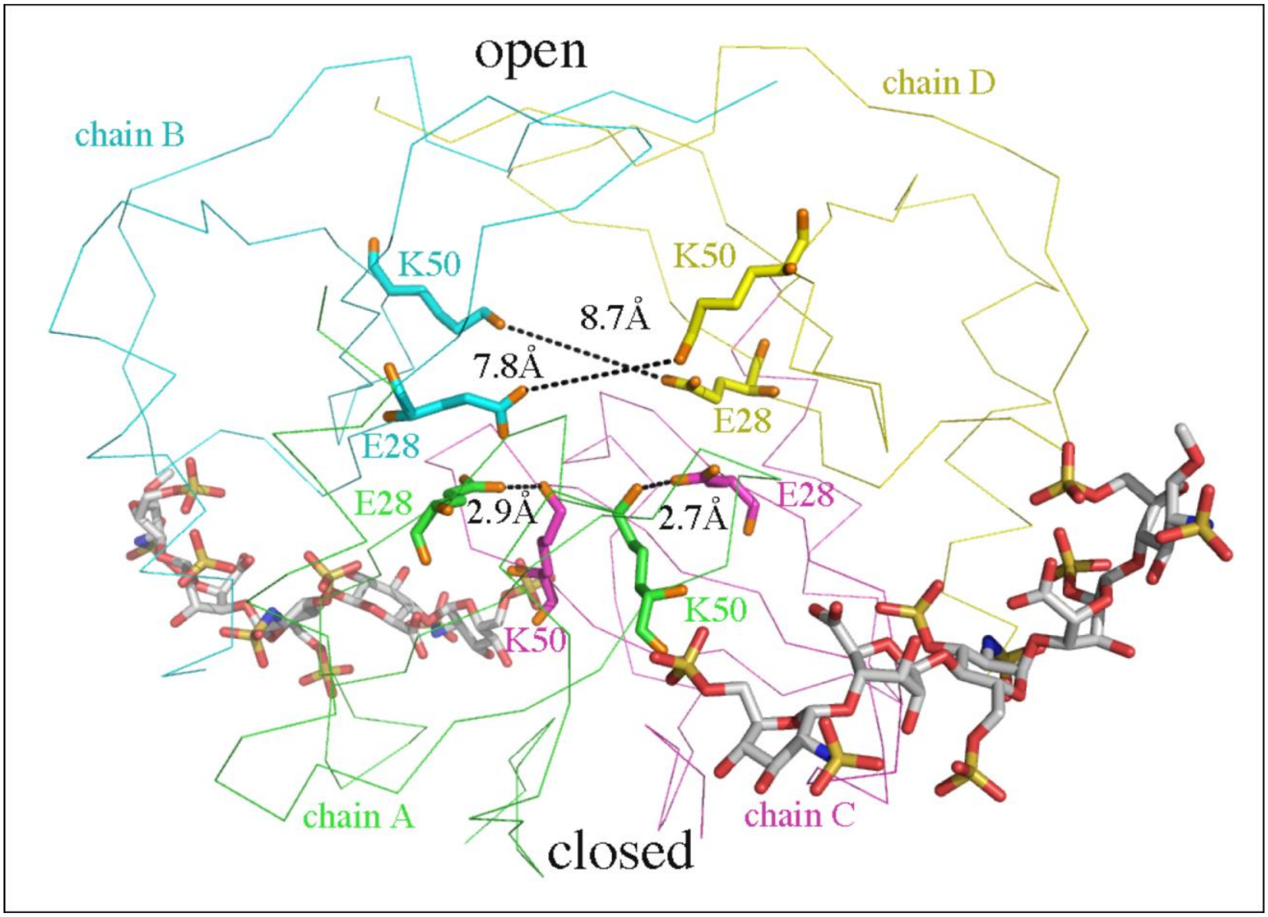

Measurement of the E28 to K50 distances shows that PF4 in the PF4/fondaparinux complex crystal adopts an asymmetric 'open'-'closed' conformation, which is almost same as in the apo-PF4 crystal structure. Salt bridges exist between chain A and chain C, not between chain B and chain D, in the asymetric tetramer. Fondaparinux binds only to the close end of PF4 tetramer and stabilizes the tetramer structure. Only two binding grooves for fondaparinux are present on the surface of the PF4 tetramer due to the asymmetry.

Supplementary Figure 3. Struture-based mutations of KKO epitopes on PF4 surface.

```
hPF4          EAEEDGDLQCLCVKTTSQ-VRPRHITSLEVIKAGPHCPTAQLIATLKNGRKICLDLQAPL 59
mPF4          PEESDGDLSCVCVKTISSGIHLKHITSLEVIKAGRHCAVPQLIATLKNGRKICLDRQAPL 60
                *.****.*:**** *. :: :*********** **...*************** ****

hPF4          YKKIIKKLLES 70
mPF4          YKKVIKKILES 71
              ***:***:***
```

Sequence alignment of human PF4 with mouse PF4 guided us to make struture-based mutations in the KKO epitope (main text Figure 2B). PF4-SCV$^{9-11}$; PF4-R^{55}.

Supplementary Figure 4. KKO recognizes the surface of the 'open' end on the PF4 in the KKOFab/PF4 complex.

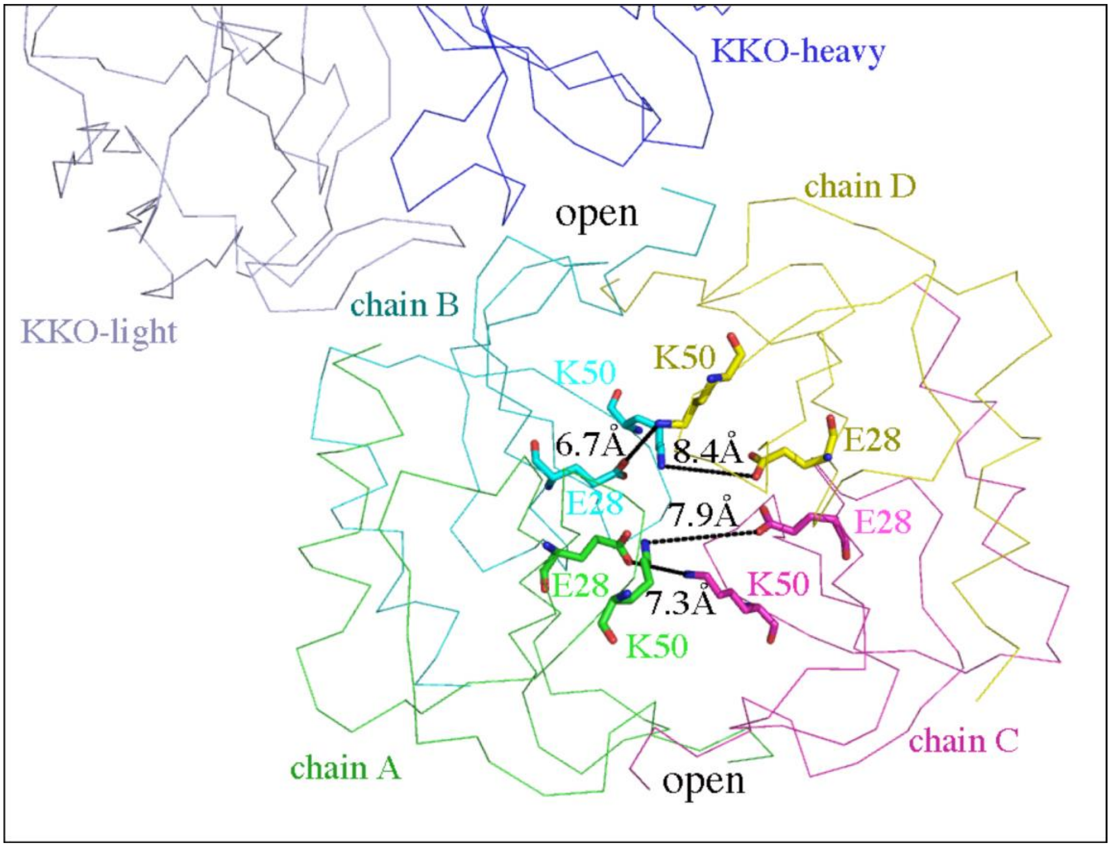

Measurement of the E28 to K50 distances (salt bridges exist between chain A and chain C, not between chain B and chain D, in the asymetric tetramer, supplemental figure 2) reveals that PF4 in the KKOFab/PF4 complex adopts a symmetric 'open'-'open' conformation. This finding provides evidence that the HIT antibody KKO recognizes the molecular surface of the 'open' end on a PF4 tetramer, which is exposed after the stabilization of the tetramer structure by a heparin.

Supplementary Figure 5. FPLC profiles of RTO-Fab/PF4 Complex.

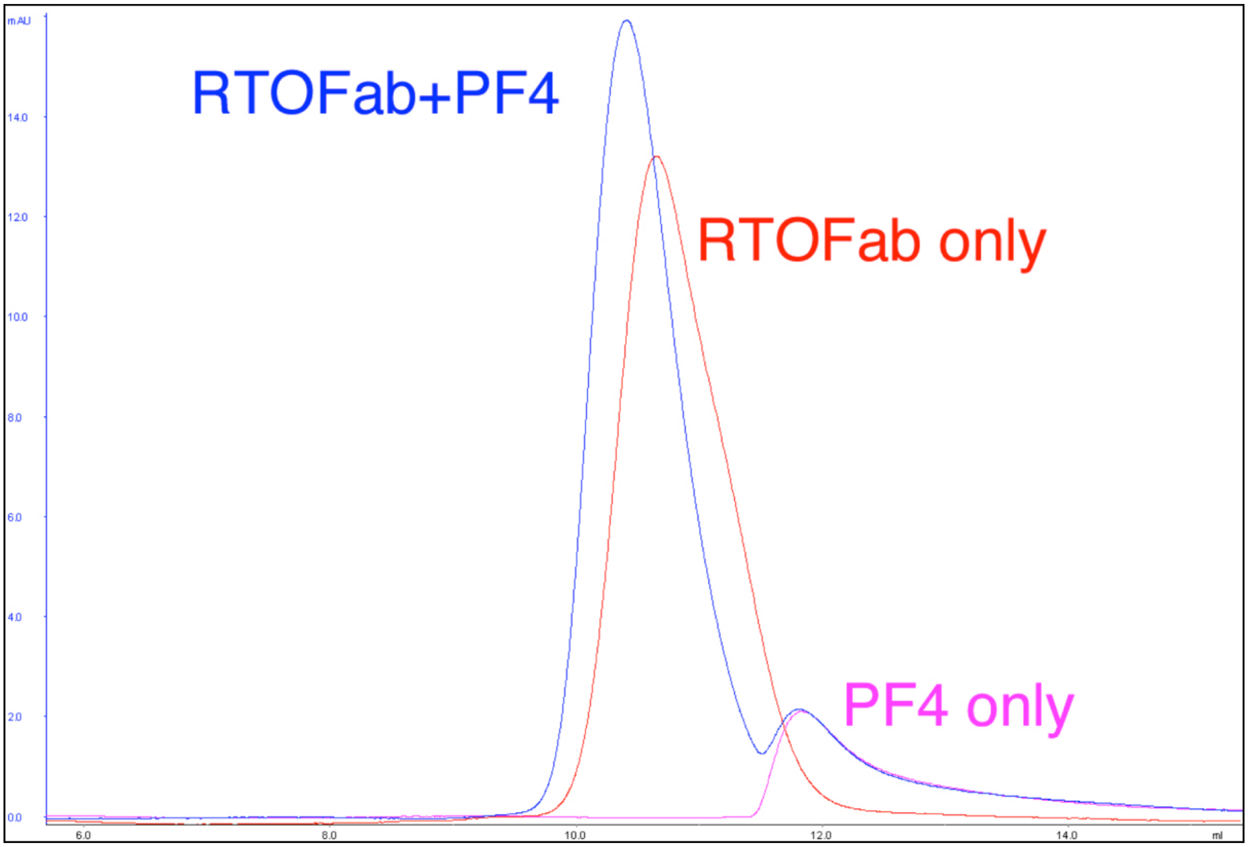

Gel filtration profiles of wild type PF4 only (magentas), RTOFab only (red) and RTOFab/PF4 complex (blue) on a Superdex 75 (GE Healthcare) column. The Superdex 75 column was calibrated using standard proteins and then molecular weight of each peak was determined. The difference between the molecular weight of the RTOFab/PF4 complex peak and that of the RTOFab only peak is about 7,000 dalton, which corresponds to the molecular weight of a PF4 monomer.

Supplementary Figure 6. Comparison of the RTO epitope on a PF4 monomer and the KKO epitope on a PF4 tetramer.

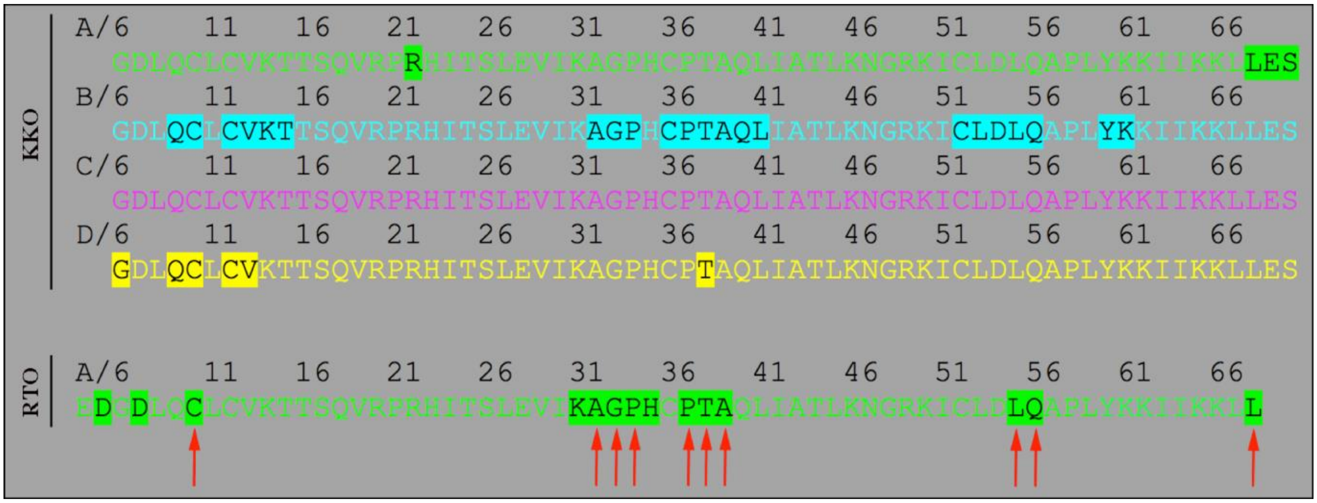

Red arrows on the bottom denote overlapping sites of interactions on PF4. The RTO epitope on PF4 overlaps with the KKO epitope, especially within the A32-A39 loop.

Supplementary Figure 7. Representative electron density maps.

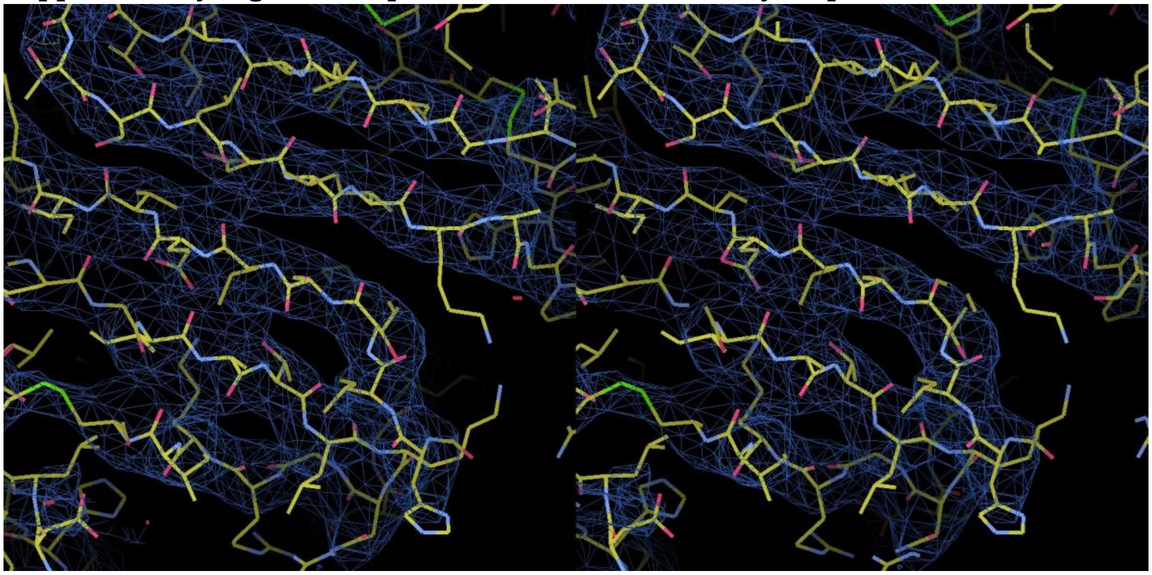

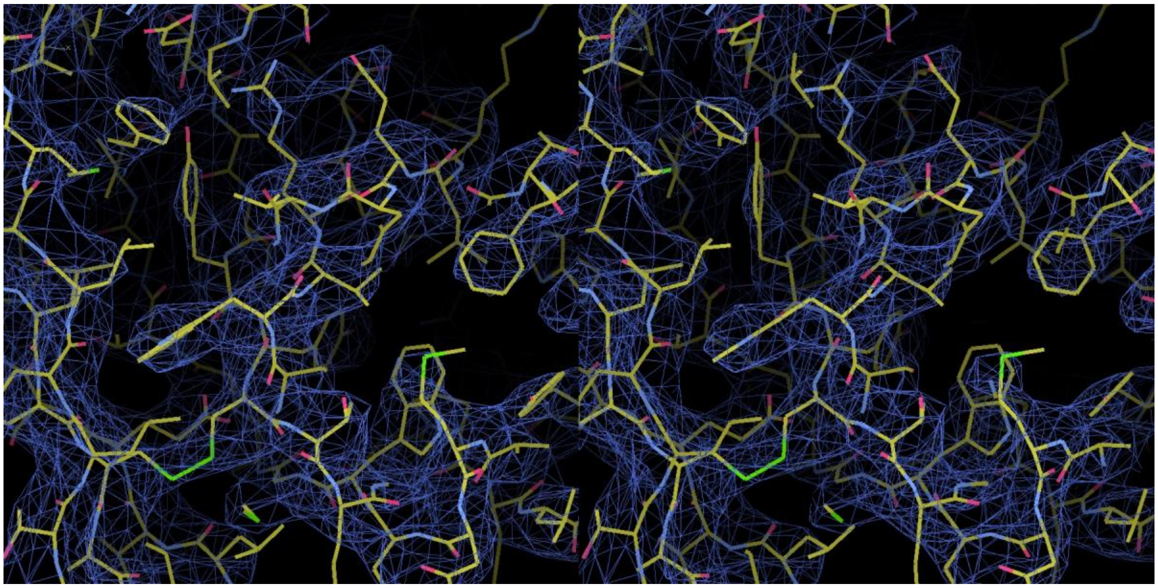

Stereo images of a portion of the 2Fo−Fc electron density map (at 1.5σ contour level and colored in blue) for the KKO-Fab/PF4 tetramer complex (up) and the 2Fo−Fc electron density map (at 1.3σ contour level and colored in blue) for the RTO-Fab/PF4 monomer complex (down).

Supplementary Table 1. Sequences of primers that were used to generate PF4 mutants.

Primers	Sequences
9SCV	GAA GAA GAT GGC GAC CTG AGC TGC GTG TGT GTG AAG ACC
9SCV-anti	GGT CTT CAC ACA CAC GCA GCT CAG GTC GCC ATC TTC TTC
55R	AGG AAA ATT TGC TTG GAC CGC CAA GCT CCG CTG TAC
55R-anti	GTA CAG CGG AGC TTG GCG GTC CAA GCA AAT TTT CCT
